# An assessment of the content of antenatal care provided by obstetricians in Lebanon: A cross-sectional study

**DOI:** 10.1371/journal.pgph.0003853

**Published:** 2024-11-04

**Authors:** Tanya El Khoury, Marie-Claire Rebeiz, Berthe Abi Zeid, Sara Mansour, George Yared, Faysal El Kak, Chaza Akik, Stephen J. McCall

**Affiliations:** 1 Faculty of Health Sciences, Center for Research on Population and Health, American University of Beirut, Beirut, Lebanon; 2 Department of Obstetrics & Gynecology, Lebanese American University, Beirut, Lebanon; 3 Faculty of Health Sciences, Department of Health Promotion and Community Health, American University of Beirut, Beirut, Lebanon; 4 Department of Obstetrics & Gynecology, American University of Beirut Medical Center, Beirut, Lebanon; PLOS: Public Library of Science, UNITED STATES OF AMERICA

## Abstract

Quality and timely antenatal care is a vital component of pregnancy care for the well-being of mothers and babies. The aim of this study of to assess self-reported obstetricians’ adherence to national and international antenatal care guidelines in Lebanon. This cross-sectional study approached all obstetricians registered with the Lebanese Society of Obstetrics and Gynecology and the Lebanese Order of Physicians to participate in an online and telephone-based survey. The study tool included all of the items from the World Health Organization and national guidelines for antenatal care. Obstetricians’ self-reported adherence was assessed across five modules including dietary interventions, dietary supplements, antenatal care assessment, fetal growth assessment, and antenatal care preventive measures. A total of 134 obstetricians completed the survey. Overall, adherence was high for most antenatal care guidelines except for providing nutrition services to undernourished pregnant women, screening for intimate partner violence, and providing tetanus vaccines. The number of recommended antenatal care visits (≥8 vs. <8 visits) was higher among obstetricians in Mount Lebanon or Beirut compared to other areas in Lebanon (72.7%vs.48.6%; p-value <0.01). Provision of tetanus or diphtheria, tetanus, and pertussis (DTP) vaccination was lower for obstetricians who provide care for women who pay out-of-pocket compared to obstetricians who provide care to women who use other payment methods (25.3%vs.45.7%; p-value = 0.024). Group B streptococcus screening test and the provision of at least five ultrasounds were higher among obstetricians who provide care in private clinics or clinics in private hospitals compared to clinics in public hospitals or primary healthcare centers (88.8%vs.66.7%; p-value = 0.023) and (83.6%vs.55.6%; p-value = 0.011), respectively. Reinforcing the adherence to all antenatal care guidelines by continuous monitoring of health facilities is crucial for best practice. Subsidies for essential services may be required for those who are unable to afford components of antenatal care.

## Introduction

Both maternal and perinatal morbidity and mortality are major public health concerns as they remain the leading causes of premature morbidity and mortality. In 2020, an estimated 287 000 women globally died from a maternal cause [1]. In 2015, there were 2.6 million neonatal deaths and 2.1 million stillbirths worldwide [2–5]. According to the World Health Organization (WHO), 94% of maternal deaths occur in low-and middle-income countries (LMICs), and most are preventable [6]. Therefore, it is critical to reduce maternal and perinatal mortality specifically in LMICs, by ensuring a safe childbirth for mother and child, which can be accomplished through access to high-quality antenatal care [7,8].

Antenatal care (ANC) is the health care that pregnant women receive from skilled healthcare professionals to ensure safe childbirth and promote the health of the mother and newborn throughout the pregnancy [9]. During ANC visits, women are provided with tests and examinations, health education and promotion to enable a healthy pregnancy [9]. ANC is an instrument that aims to detect and treat pregnancy complications and identify women at high risk of complications during childbirth [9]. The WHO initiated evidence-based guidelines for ANC to reduce perinatal morbidity and mortality through improving the quality of care received. Although global coverage of early initiation of ANC and attendance of at least four ANC visits has increased, inequalities in coverage persist [10,11], as well as large variation in the content and quality of care provided [12].

The WHO released comprehensive ANC recommendations in various areas for pregnant women and adolescent girls [9]. These evidence-based guidelines aim to provide good quality and appropriate ANC with a minimum of eight visits between conception and birth [9]. However, these are general, non-setting specific guidelines that need to be adapted and contextualized. In 2016, the United Nations Population Fund (UNFPA)-Lebanon office in collaboration with the Ministry of Public Health (MOPH) released ANC guidelines as part of the service delivery guidelines to be implemented and utilized at primary health care level; yet, it remains unclear to what extent these guidelines are adopted by obstetricians in Lebanon [13].

Studies have shown that high ANC coverage does not necessarily translate into adequate content and high quality of care, particularly in LMICs [12,14]. Hence, adherence of health providers to ANC guidelines is a vehicle for uniformity of clinical care and a component of quality of care [15]. In Lebanon, studies on ANC are limited. A previous study in 2010 showed that infrastructure and human resources are key components for high coverage of ANC in Lebanon [16]. Other recent studies have focused on Syrian refugees, which showed limited uptake of ANC [17–19]. As for studies conducted on the Lebanese population regarding ANC guidelines adherence, the most recent was conducted in 2004 [20], and currently requires updating according to new practices and recommendations [16]. Therefore, a more comprehensive picture of ANC provision in Lebanon is still needed.

There is paucity of data about the quality and content of ANC provided in Lebanon. One component of quality of care is obstetricians’ adherence to ANC guidelines, which reflects the content of care provided during each visit. Measuring obstetricians’ adherence to the updated WHO and MOPH ANC guidelines can provide an understanding of existing gaps in care. This study aims to describe the provision of ANC in Lebanon using existing national and international guidelines, and whether adherence differs by type of healthcare facility, area, and primary payment method.

## Methods

### Ethics statement

This study was approved by Social and Behavioral Sciences Institutional Review Board (IRB) of the institution (SBS-2021-0037). Consent to participate was obtained verbally from all participants for this telephone survey. The consent process and obstetricians’ responses were documented on Limesurvey platform. The IRB approved the use of verbal consent for this study.

### Study design and setting

This was a cross-sectional study conducted in collaboration with the Lebanese Society of Obstetrics and Gynecology (LSOG) to assess adherence to ANC guidelines amongst obstetricians registered with the Lebanese Order of Physicians and LSOG in Lebanon. This study followed the Strengthening the Reporting of Observational Studies in Epidemiology (STROBE) reporting guidelines [21].

### Study population

Obstetricians are the main source of ANC provision in Lebanon; therefore, nurses, midwives, and other healthcare professionals were not interviewed. Only obstetricians who worked in a healthcare facility for at least six months were eligible for the study. Those who retired, left the country, did not work in obstetrics, were not available, unreachable, or refused to participate were excluded from the study.

### Sample selection

Between November 2021 and March 2022, obstetricians who were registered with the LSOG and Lebanese Order of Physicians were approached to participate in this study by email and through telephone invitation. An initial email invitation that included a link to the survey was sent to the registered obstetricians, while giving them the choice to complete the questionnaire electronically (self-administered) or via phone call interview. If after a month there was no response, obstetricians were contacted via telephone using phone numbers provided by the LSOG and Lebanese Order of Physicians. A clinically trained member of the team (MCR) undertook computer-assisted telephone interviews using the Limesurvey platform.

### Data collection tool

A comprehensive list of components of care was mapped and drafted into a questionnaire using the existing WHO guidelines and the MOPH service delivery guidelines for ANC [9,13]. The WHO and MOPH guidelines were compiled together. If MOPH included a guideline that was not included in the WHO compilation, it was added. The survey included closed questions to determine the content of care for each item in the guidelines. The tool was based on five key modules: maternal and fetal growth assessment, antenatal care assessment, dietary interventions, dietary supplements, and ANC preventive measures. The dietary interventions module was divided into two sections: the first was addressed to obstetricians who provide care to pregnant women, and the second was related to those who provide care to undernourished pregnant women. Within each module, a question was asked per guideline item to determine whether the component of care was provided. In addition, the questionnaire also gathered general information about the type of healthcare facility, location, and primary method of payment used by women seeking ANC (social security, MOPH, out-of-pocket or private insurance, UNHCR or UNRWA). Given that some obstetricians practiced in more than one location (for example, private clinic vs. primary healthcare center), they were asked to complete the questionnaire based on the healthcare facility where they spent most of their time. The tool was reviewed by academics and two obstetricians before translating it into Arabic. Prior to onset of data collection, the English and Arabic versions of the questionnaires were piloted internally.

### Outcome measures

Obstetricians’ adherence to ANC guidelines was determined based on the responses documented by the data collector, who was a clinically trained physician, on whether each of the recommended services, tests or advice were implemented during ANC visits (Yes/No). The interviewer asked the obstetricians whether they performed each item related to the ANC visit as proposed by the international and national guidelines. Therefore, a score of 1 was given for the obstetricians’ responses that aligned with the ANC guidelines of the WHO and the MOPH, and a score of 0 was given if they did not. The items were grouped into five modules that included fetal growth assessment (8 items), dietary interventions (8 items: 5 items for the first section, and 3 items for the second section), dietary supplements (8 items), ANC assessment (15 items), and ANC preventive measures (9 items). For every respondent, an overall score and 5 sub-scores for each module were generated by summing up the item scores. Since the dietary intervention module was divided into two sections, an additional sub-score was generated for obstetricians who provide care to undernourished pregnant women. More details about the items of each module can be found in S1 Table. A standard linear transformation was performed to convert the obtained scores within the range of 0 to 100. Based on clinical consensus, we created a sub-set of selected ANC guideline items from every module that were considered to be assessed against facility types, areas, and payment methods.

### Statistical analysis

Frequencies and percentages were reported for categorical variables. Continuous variables were assessed for normality using histograms and the Shapiro-Wilk test. Normally distributed variables were represented as a mean with standard deviation (SD), while non-normally distributed variables were represented as median with interquartile ranges (IQR). Chi-square tests and Fischer’s exact tests were used to assess the difference of adherence to ANC across healthcare facility types, areas, and primary payment methods. Man-Whitney U tests were used to assess for the difference in scores across healthcare facility types. Partially completed questionnaires were excluded from data analysis. All analyses were conducted using Stata/SE 17.

## Results

### Characteristics of the population

Out of 1,090 obstetricians registered with the LSOG and Lebanese Order of Physicians, 80 were retired, left the country, did not work in obstetrics, or were not available, 373 refused to participate, 402 were unreachable, and 101 partially completed the questionnaire (S1 Fig). A total of 134 obstetricians completed the ANC questionnaire and were included in this study. More than half of the obstetricians (64.9%) spent most of their time providing ANC in a private clinic. Most of the obstetricians reported that their primarily place of work was in healthcare facilities located in Beirut (38.1%) and Mount Lebanon (35.8%). The most common payment method for ANC services in healthcare facilities was out-of-pocket (53.7%), and the majority of patients in most clinics were Lebanese (83.6%) (**Table 1**).

**Table 1:** Background characteristics of obstetricians (n = 134).

	n	(%)
**Type of healthcare facility where most of the time was spent**		
Primary healthcare center	17	(12.7)
Private doctor’s clinic	87	(64.9)
Clinic in public hospital	1	(0.7)
Clinic in private hospital	29	(21.6)
**Antenatal care type of payment**		
Ministry of Public Health	3	(2.2)
Military schemes	2	(1.5)
Non-Governmental Organizations (local or international)	8	(6.0)
National Social Security Fund (NSSF)	25	(18.7)
Out-of-Pocket	72	(53.7)
Private insurance	17	(12.7)
UN agencies–UNHCR	7	(5.2)
**Most common nationality for women seeking antenatal care**		
Lebanese	112	(83.6)
Syrian	21	(15.7)
Palestinian	1	(0.7)
**Governorate of practice**		
Baalbak-Hermel	3	(2.2)
Beirut	51	(38.1)
Beqaa	11	(8.2)
Mount Lebanon	48	(35.8)
Nabatieh	6	(4.5)
North	2	(1.5)
South	13	(9.7)

### Adherence to ANC guidelines

Table 2 represents adherence to ANC standards according to the national and international guidelines. Five modules were used to assess overall adherence among obstetricians. For the dietary intervention module, adherence to providing nutrition education and protein dietary supplements was 88.6% and 86.4% respectively; however, adherence to providing balanced energy and protein dietary supplements was relatively low, with only 27.3% adherence. Most obstetricians counselled about having a healthy diet (90.3%), physical activity (72.4%), excess weight during pregnancy (93.3%), and reducing caffeine intake (94%). For the dietary supplement’s module, most obstetricians recommended the intake of vitamins and minerals such as iron (89.5%), folic acid during pre-conception and the first trimester (91.8%), and calcium (63.4%). For the ANC assessment module, almost all obstetricians screened for anemia (99.3%), bacteriuria (99.3%), hyperglycemia (94.8%), gestational diabetes (92.1%), smoking (92.5%), hypertension (94.8%), requested full blood count (97.8%), midstream urine flow test (90.3%), group B streptococcus (85.8%), Toxoplasmosis, Rubella, and Hepatitis B surface antigen immunity status (95.5%), and measured blood pressure (97.8%). A smaller proportion of obstetricians screened for intimate partner violence (29.9%), substance misuse (42.5%), HIV and syphilis (51.5%), and other sexually transmitted infections (41.8%). For the fetal growth assessment module, 39.6% of obstetricians assessed fetal growth and well-being using ANC cardiotocography (non-stress test) and 48.5% by routine Doppler ultrasound. Almost all obstetricians assessed fetal growth using ultrasound scans throughout pregnancy (98.5%) (median: 7 ultrasounds [IQR: 5–9]), with the first ultrasound scan performed before 14 weeks of gestation (96.3%) and the last one at 37 weeks or after (62.7%). For the ANC preventive measures module, 91% prescribed antibiotics for bacteriuria when indicated. More than two thirds provided the flu vaccine (71.6%) and COVID-19 vaccine (84.3%). Most obstetricians gave anti-D immunoglobulin between 28 and 34 weeks of gestation (76.9%). Nearly a third of obstetricians provided the tetanus or diphtheria, tetanus, pertussis (DTP) vaccine during ANC (30.6%).

**Table 2:** Self-reported obstetricians’ adherence to items from antenatal care guidelines.

Intervention	Recommendation according to international^1^ and national guideline^2^	Obstetricians’ adherence to items			
		No		Yes	
		n	(%)	n	(%)
**Module 1 (a): Dietary interventions (n = 134) **					
**Do you provide counselling about?**	** **				
Healthy diet	Recommended	13	(9.7)	121	(90.3)
Increased physical activity	Recommended	37	(27.6)	97	(72.4)
Excess weight gain during pregnancy	Recommended	9	(6.7)	125	(93.3)
At least one of the above	Recommended	1	(0.7)	133	(99.3)
**Do you provide advice about daily caffeine intake?**	Recommended^3^	8	(6.0)	126	(94.0)
**Module 1 (b): Dietary interventions (Among those who provide care to undernourished pregnant women) (n = 44)**			** **	** **	** **
**Do you provide antenatal care about?**					
Nutrition education	Recommended	5	(11.4)	39	(88.6)
Balanced energy and protein dietary supplements	Recommended	32	(72.7)	12	(27.3)
High protein supplements	Not recommended	38	(86.4)	6	(13.6)
**Module 2: Dietary supplements (n = 134)**					
**Do you recommend dietary supplementation such as vitamins and minerals?**	Recommended	7	(5.2)	127	(94.8)
**Type of supplementation recommended**					
Iron	Recommended	14	(10.5)	120	(89.5)
Folic acid	Recommended during pre-conception and first trimester	11	(8.2)	123	(91.8)
Calcium	Recommended if woman has low dietary calcium intake	49	(36.6)	85	(63.4)
Multiple micronutrients	Not recommended	58	(43.3)	76	(56.7)
Vitamin B6	Not recommended	32	(23.9)	102	(76.1)
Vitamin E and C	Not recommended	16	(11.9)	118	(88.1)
Vitamin D	Not recommended	36	(26.9)	98	(73.1)
**Module 3: Antenatal care assessment (n = 134)**					
**Do you screen for any of the following risk factors or conditions?**					
Anemia	Recommended	1	(0.7)	133	(99.3)
Bacteriuria	Recommended	1	(0.7)	133	(99.3)
Intimate partner violence	Recommended	94	(70.1)	40	(29.9)
Hyperglycemia	Recommended	7	(5.2)	127	(94.8)
Smoking during pregnancy	Recommended	10	(7.5)	124	(92.5)
Substance misuse	Recommended	77	(57.5)	57	(42.5)
HIV and Syphilis	Recommended	65	(48.5)	69	(51.5)
Hypertension	Recommended	7	(5.2)	127	(94.8)
Other sexually transmitted infections	Recommended	78	(58.2)	56	(41.8)
**Do you provide any of the following tests during the ANC period?**					
Full blood count	Recommended	3	(2.2)	131	(97.8)
If no to full blood count, other haemoglobin testing	Recommended if no full blood count	0		3	(100.0)
Midstream urine flow	Recommended	13	(9.7)	121	(90.3)
If no to midstream urine flow, dipstick testing	Recommended if no to midstream urine flow	5	(38.5)	8	(61.5)
Group B streptococcus screening testing	Recommended	19	(14.2)	115	(85.8)
Immunity status check for Toxoplasmosis, Rubella and Hepatitis B surface antigen	Recommended	6	(4.5)	128	(95.5)
Blood pressure measurements	Recommended	3	(2.2)	131	(97.8)
None of the above recommended tests	Not recommended	0		134	(100.0)
**Do you screen your patients for gestational diabetes by ordering the 1-hour glucose challenge test?**	Recommended	24	(17.9)	110	(92.1)
**Module 4: Fetal growth assessment (n = 134)**					
**Do you assess fetal growth and well-being with any of the following measures?**					
Recommend fetal movement chart	Recommended based on the context	72	(53.7)	62	(46.3)
Measure symphysis-fundal height	Not recommended	38	(28.4)	96	(71.6)
Assess fetal growth via abdominal palpation	Not recommended	34	(25.4)	100	(74.6)
Perform antenatal cardiotocography	Recommended	81	(60.4)	53	(39.6)
Perform routine Doppler ultrasound	Recommended	69	(51.5)	65	(48.5)
Perform ultrasound	Recommended	2	(1.5)	132	(98.5)
None of the above recommended measures	Not recommended	0		134	(100.0)
**Number of ultrasounds performed** [n = 132; Median (IQR)]	At least one scan before 24 weeks of gestation, then at least 1 scan every month			7	(5–9)
Performance of at least 5 ultrasounds during pregnancy		27	(20.1)	107	(79.9)
**Performance of the first ultrasound during the first trimester**	Recommended during the first trimester	5	(3.7)	129	(96.3)
**Performance of the last ultrasound after 37 weeks of gestation**	Recommended until delivery	50	(37.3)	84	(62.7)
**Module 5: Antenatal care preventive measures (n = 134)**					
**Do you provide any of the following medications/treatments during antenatal care?**					
Antibiotics when indicated	Recommended when indicated	3	(2.2)	131	(97.8)
Antibiotics for group B streptococcus infection	Recommended when indicated	44	(32.8)	90	(67.2)
Antibiotics for bacteriuria	Recommended when indicated	12	(9.0)	122	(91.0)
Antibiotics to prevent recurrent urinary tract infection	Not recommended	67	(50.0)	67	(50.0)
Tetanus vaccine or DTP^4^	Recommended	93	(69.4)	41	(30.6)
Flu vaccine	Recommended	38	(28.4)	96	(71.6)
For women at risk, pre-exposure prophylaxis to prevent HIV	Recommended based on the context	117	(87.3)	17	(12.7)
COVID-19 vaccine	Recommended	21	(15.7)	113	(84.3)
None of the above recommended medications/treatments^5^	Not recommended	3	(2.2)	131	(97.8)
**Do you give anti-D immunoglobulin between 28 and 34 weeks of gestation?**	Recommended	31	(23.1)	103	(76.9)

1: World Health Organization.

2: Lebanese Ministry of Public Health.

3: Adapted, since obstetricians who gave advice regarding caffeine intake, regardless of the amount, are considered to have adhered to the recommendations.

4: DTP: Diphtheria, tetanus, and pertussis vaccination.

5: Including antibiotics in general when indicated, tetanus vaccine or DTP, flu vaccine and pre-exposure prophylaxis to prevent HIV.

### Adherence to ANC guidelines by healthcare facility type, area, and payment method

Table 3 shows the distribution of selected ANC services according to the area where the healthcare facility is located (Mount Lebanon or Beirut vs. Other). The provision of flu vaccine, COVID-19 vaccine, full blood count, group B streptococcus screening test, blood pressure measurements, midstream urine flow test, five or more ultrasound scans, iron and folic acid supplements, and gestational diabetes screening was high across all areas with no statistical difference. The number of ANC visits (≥8 vs. <8 visits) recommended to pregnant women was significantly higher among providers located in Mount Lebanon or Beirut compared to those located in other areas of the country (72.7% vs. 48.6%; p-value <0.01). The provision of group B streptococcus screening test (88.8% vs. 66.7%; p-value = 0.023) and performing at least 5 ultrasounds during pregnancy (83.6% vs. 55.6%; p-value = 0.011) were significantly higher among obstetricians practicing in private clinics or clinics in private hospitals compared to those practicing in clinics in public hospitals or primary healthcare centers. The provision of other ANC items was similar with no significant difference across different facility types (Table 4). Adherence to the selected ANC items was high with no significant difference across different payment methods (out-of-pocket vs. other payment methods), except for the provision of the tetanus or diphtheria, tetanus, and pertussis (DTP) vaccine; the latter was significantly lower among obstetricians taking care of women who pay out-of-pocket compared to those who use other payment methods (25.3% vs. 45.7%; p-value = 0.024) (S2 Table).

**Table 3:** Adherence to selected antenatal care services by area in Lebanon.

	Mount Lebanon or Beirut (n = 99 (73.9%))		All other areas in Lebanon* (n = 35 (26.1%))		P-value
	n	(%)	n	(%)	
**Do you provide tetanus vaccine or DTP?**					
No	68	(68.7)	25	(71.4)	0.762
Yes	31	(31.3)	10	(28.6)	
**Do you provide flu vaccine?**					
No	27	(27.3)	11	(31.4)	0.639
Yes	72	(72.7)	24	(68.6)	
**Do you provide COVID-19 vaccine?**					
No	18	(18.2)	3	(8.6)	0.179
Yes	81	(81.8)	32	(91.4)	
**Do you provide full blood count?**					
No	2	(2.0)	1	(2.9)	1.000^f^
Yes	97	(98.0)	34	(97.1)	
**Do you provide group B streptococcus screening test?**					
No	11	(11.1)	8	(22.9)	0.087
Yes	88	(88.9)	27	(77.1)	
**Do you provide blood pressure measurements?**					
No	3	(3.0)	0	(0.0)	0.567^f^
Yes	96	(97.0)	35	(100.0)	
**Do you provide midstream urine flow test?**					
No	11	(11.1)	2	(5.7)	0.513^f^
Yes	88	(88.9)	33	(94.3)	
**Do you perform at least 5 ultrasounds during pregnancy?**					
No	22	(22.2)	5	(14.3)	0.314
Yes	77	(77.8)	30	(85.7)	
**Do you recommend iron?**					
No	10	(10.1)	4	(11.4)	0.759^f^
Yes	89	(89.9)	31	(88.6)	
**Do you recommend folic acid during pre-conception and the first trimester?**					
No	8	(8.1)	3	(8.6)	1.000^f^
Yes	91	(91.9)	32	(91.4)	
**Do you screen your patients for gestational diabetes by ordering the 1-hour glucose challenge test?**					
No	18	(18.2)	6	(17.1)	0.890
Yes	81	(81.8)	29	(82.9)	
**Number of visits recommended**					
Less than 8 visits	27	(27.3)	18	(51.4)	0.009
8 or more visits	72	(72.7)	17	(48.6)	
**Adherence to guidelines**					
Adherence to all	15	(14.1)	1	(2.9)	0.115^f^
Adherence to some	85	(85.9)	34	(97.1)	

*Other areas include: Baalbak-Hermel, Beqaa, Nabatieh, North and South.

^f^ Fisher’s exact test was used.

**Table 4:** Adherence to selected antenatal care services by healthcare facility type in Lebanon.

	Clinic in private hospital or private doctor clinic (n = 116 (86.6%))		Clinic in public hospital or primary healthcare center (n = 18 (13.4%))		P-value
	n	(%)	n	(%)	
**Do you provide tetanus vaccine or DTP?**					
No	82	(70.7)	11	(61.1)	0.412
Yes	34	(29.3)	7	(38.9)	
**Do you provide flu vaccine?**					
No	32	(27.6)	6	(33.3)	0.615
Yes	84	(72.4)	12	(66.7)	
**Do you provide COVID-19 vaccine?**					
No	19	(16.4)	2	(11.1)	0.738^f^
Yes	97	(83.6)	16	(88.9)	
**Do you provide full blood count?**					
No	2	(1.7)	1	(5.6)	0.354^f^
Yes	114	(98.3)	17	(94.4)	
**Do you provide group B streptococcus s test?**					
No	13	(11.2)	6	(33.3)	0.023^f^
Yes	103	(88.8)	12	(66.7)	
**Do you provide blood pressure measurements?**					
No	3	(2.6)	0	(0.0)	1.000^f^
Yes	113	(97.4)	18	(100.0)	
**Do you provide midstream urine flow test?**					
No	11	(9.5)	2	(11.1)	0.687^f^
Yes	105	(90.5)	16	(88.9)	
**Do you perform at least 5 ultrasounds during pregnancy?**					
No	19	(16.4)	8	(44.4)	0.011^f^
Yes	97	(83.6)	10	(55.6)	
**Do you recommend iron?**					
No	12	(10.3)	2	(11.1)	1.000^f^
Yes	104	(89.7)	16	(88.9)	
**Do you recommend folic acid during pre-conception and the first trimester?**					
No	8	(6.9)	3	(16.7)	0.168^f^
Yes	108	(93.1)	15	(83.3)	
**Do you screen your patients for gestational diabetes by ordering the 1-hour glucose challenge test?**					
No	21	(18.1)	3	(16.7)	1.000^f^
Yes	95	(81.9)	15	(83.3)	
**Number of visits recommended**					
Less than 8 visits	37	(31.9)	8	(44.4)	0.294
8 or more visits	79	(68.1)	10	(55.6)	
**Adherence to guidelines**					
Adherence to all	14	(12.1)	1	(5.6)	0.692^f^
Adherence to some	102	(87.9)	17	(94.4)	

^f^ Fisher’s exact test was used.

### Score of ANC adherence for each domain

A score of adherence was generated for the five modules of the questionnaire. Overall (out of 45 items), the adherence score ranged between 42.2% and 95.6% with a median score of 77.8% [IQR: 68.9%; 80%]. Among all obstetricians who provide care for pregnant women, the highest level of adherence to ANC guidelines was observed for the dietary interventions (median: 100% [IQR: 80%; 100%]), followed by dietary supplements (median: 87.5% [IQR: 75%; 87.5%]). A slightly lower adherence was observed in the domains of antenatal care assessment (median: 80% [IQR: 73.3%; 86.7%]) and fetal growth assessment (median: 75% [IQR: 62.5%; 87.5%]). The lowest adherence score was observed for the antenatal care preventive measures (median: 66.7% [IQR: 55.6%; 77.8%]). In addition, among obstetricians who provided care for undernourished pregnant women, the adherence to items related to dietary interventions or counselling for these women was 66.7% (Table 5). The adherence scores did not differ significantly across healthcare facility types, areas, and payment methods (S3 Table).

**Table 5:** Overall and module-specific score of obstetricians’ adherence to antenatal care guidelines.

	Actual scores			Linear transformation (%)		
	Range	Median	[IQR]	Range	Median	[IQR]
**Overall score (n = 134)**	19; 43	35	[31; 36]	42.2; 95.6	77.8	[68.9; 80]
**Sub score for all respondents (n = 134)**						
Dietary interventions	1; 5	5	[4; 5]	20; 100	100	[80; 100]
Dietary supplements	0; 8	7	[6; 7]	0; 100	87.5	[75; 87.5]
Antenatal care assessment	7; 15	12	[11; 13]	46.7; 100	80.0	[73.3; 86.7]
Fetal growth assessment	0; 8	5	[4; 6]	0; 100	75.0	[62.5; 87.5]
Antenatal care preventive measures	0; 8	6	[5; 7]	0; 88.9	66.7	[55.6; 77.8]
**Sub score for respondents who provide care to undernourished pregnant women (n = 44)**						
Dietary interventions	0; 3	2	[2; 2]	0; 100	66.7	[66.7; 66.7]

## Discussion

This study on self-reported adherence showed that there was a moderate to high level of obstetricians’ adherence to ANC guidelines with no difference in scores across healthcare facility type, areas, and payment methods. Performing at least five ultrasounds during pregnancy and provision of group B streptococcus screening was higher among obstetricians who practice in private clinics or clinics in private hospitals compared to those who practice in clinics in public hospitals or primary health care settings. Nearly a third of the obstetricians adhered to the provision of tetanus or DTP vaccination, and this was lower among those who have more patients who paid out-of-pocket.

### Results in context

Adherence to ANC preventive measures score was acceptable among obstetricians, where half of the participants had a score between 55.6% and 77.8%. However, adherence of obstetricians to the provision of tetanus or DTP vaccine, flu vaccine, and COVID-19 vaccine was low. Vaccines are an essential component of care that can prevent maternal and perinatal morbidity [22–24]. With regards to the tetanus or DTP vaccine, the observed percentages were lower than those reported in LMICs such as Ethiopia and Tanzania with rates of adherence above 85% [25,26]. Strengthening the adherence of obstetricians to providing tetanus vaccination through regular monitoring and training on the importance of vaccine provision is necessary to prevent adverse pregnancy outcomes and to reach optimal obstetric care.

The reported average number of ANC visits was significantly less than eight among obstetricians who practice in areas outside Beirut and Mount Lebanon compared to those who practice in Beirut and Mount Lebanon. According to the recent WHO and the UNFPA-Lebanon guidelines, a minimum of eight ANC visits should be provided to improve the quality and content of care. The results might be related to women’s circumstances rather than obstetricians, as many women struggle to access ANC in LMICs, particularly in rural areas [11,27]. Notably, pregnant women in rural areas in Lebanon may have low socioeconomic characteristics, which may limit the number of ANC visits due to financial reasons. Under-attendance (1–5 visits) is associated with adverse health outcomes [28]. Hence, it is advisable for municipalities in each governorate to collaborate with international organizations to provide educational and financial support to local community clinics, healthcare dispensaries and public health departments. This collaboration would ensure the availability of accessible and affordable ANC services.

The provision of five or more ultrasounds during pregnancy was higher among obstetricians practicing in private practice compared to those practicing in clinics in public hospitals. This difference could be attributed to the socioeconomic status of women seeking ANC. Additionally, the financial strain caused by the economic crisis in Lebanon may have further hindered the ability to afford regular check-ups and led to fewer ultrasounds in public hospitals settings.

The lowest score of adherence was observed for the dietary intervention domain related to undernourished pregnant women, particularly in providing balanced energy and protein dietary supplementation. Although studies targeting adherence among undernourished pregnant women are limited, our results are aligned with a recent study conducted among pregnant women in Bangladesh, where low adherence to nutrition services by healthcare providers was reported [29]. This could be explained by obstetricians prioritizing curative health services over preventive nutritional services due to high workload pressure [29]. Previous studies have shown that provision of nutritional education to undernourished pregnant women and enabling women to have a balanced diet reduced maternal complications, stillbirth, and infant low birth weight [30–32]. In Lebanon, a large proportion of the population has fallen below the poverty line [33], which has exacerbated pre-existing food insecurity rates [34], therefore, nutritional interventions and counselling remain an essential component of ANC in the Lebanese context.

### Recommendations

By prioritizing and strengthening adherence to the essential components of ANC, we can reduce the risk of maternal and fetal complications and promote healthier outcomes for both mothers and infants. Hence, ongoing professional development and training programs tailored to the specific needs of obstetricians would enhance adherence and enable the delivery of comprehensive ANC. In addition, subsidies for crucial components of ANC should be provided to all women in Lebanon to tackle underlying inequalities. Future qualitative research would provide valuable insights into the perspectives and experiences of pregnant women, obstetricians, and other stakeholders involved in ANC provision. In addition, future studies should consider cross-referencing obstetricians’ responses with electronic health records or patients’ surveys.

### Strength and limitations

This study addresses an important knowledge gap in obstetricians’ adherence to ANC services in Lebanon and presents a comprehensive score using the updated WHO and national guidelines. In addition, this study considered multi-level factors (healthcare facility type, area, and payment method) and applied a stratified descriptive analysis to target recommendations to improve service delivery.

This study is subject to several limitations. The outcomes were self-reported and were not cross-referenced with other sources such as electronic medical records, which may introduce information bias. The small sample size may not be representative of all the LSOG community so the findings cannot be generalized. In addition, there is a possibility that a proportion of obstetricians with busy practices were not available to participate in the study. Furthermore, factors that could influence the provision of ANC services were not explored in this study including resource availability in healthcare facilities and obstetricians’ workload. Additionally, we did not collect certain background characteristics of the obstetricians, such as years of experience, age, and other factors.

## Conclusions

The present study underlined the importance of reinforcing the adherence to all ANC guidelines for the best possible practice. Overall, high adherence to multiple components of ANC was noted. However, there was low adherence in the provision of nutritional counselling and interventions to undernourished pregnant women, and also in the provision of tetanus or DTP vaccination. Furthermore, disparities in provision of ANC are required to be tackled in Lebanon. Future research is needed to identify factors influencing the provision of ANC from both the obstetricians’ and women’s perspectives for better intervention planning.

## Supporting information

S1 Fig Flow diagram of study sample selection. (TIFF)

S1 Table Items used to generate adherence score. (XLSX)

S2 Table Adherence of selected antenatal care services by different payment methods in Lebanon. (DOCX)

S3 Table Overall and module-specific score of obstetricians’ adherence by area, payment method, and healthcare facility type. (DOCX)

## References

[1] World Health Organization. Trends in maternal mortality 2000 to 2020: estimates by WHO, UNICEF, UNFPA, World Bank Group and UNDESA/Population Division: executive summary. 2023. Report No.: 9240069259.

[2] Wang H, Abajobir AA, Abate KH, Abbafati C, Abbas KM, Abd-Allah F, et al. Global, regional, and national under-5 mortality, adult mortality, age-specific mortality, and life expectancy, 1970–2016: a systematic analysis for the Global Burden of Disease Study 2016. The Lancet. 2017;390(10100):1084–150. doi: 10.1016/S0140-6736(17)31833-0 28919115 PMC5605514

[3] Kassebaum NJ, Barber RM, Bhutta ZA, Dandona L, Gething PW, Hay SI, et al. Global, regional, and national levels of maternal mortality, 1990–2015: a systematic analysis for the Global Burden of Disease Study 2015. The Lancet. 2016;388(10053):1775–812. 10.1016/S0140-6736(16)31470-2 PMC5224694 27733286

[4] Blencowe H, Cousens S, Jassir FB, Say L, Chou D, Mathers C, et al. National, regional, and worldwide estimates of stillbirth rates in 2015, with trends from 2000: a systematic analysis. The Lancet Global Health. 2016;4(2):e98–e108. doi: 10.1016/S2214-109X(15)00275-2 26795602

[5] Kuruvilla S, Bustreo F, Kuo T, Mishra C, Taylor K, Fogstad H, et al. The Global strategy for women’s, children’s and adolescents’ health (2016–2030): a roadmap based on evidence and country experience. Bulletin of the World Health Organization. 2016;94(5):398. doi: 10.2471/BLT.16.170431 27147772 PMC4850541

[6] World Health Organization. Maternal mortality. 2019 [Available from: https://www.who.int/news-room/fact-sheets/detail/maternal-mortality.

[7] Carroli G, Rooney C, Villar J. How effective is antenatal care in preventing maternal mortality and serious morbidity? An overview of the evidence. Paediatric and perinatal Epidemiology. 2001;15:1–42. doi: 10.1046/j.1365-3016.2001.0150s1001.x 11243499

[8] Villar J, Bergsjø P. Scientific basis for the content of routine antenatal care I. Philosophy, recent studies, and power to eliminate or alleviate adverse maternal outcomes. Acta obstetricia et gynecologica Scandinavica. 1997;76(1):1–14. 9033238 10.3109/00016349709047778

[9] World Health Organization. WHO recommendations on antenatal care for a positive pregnancy experience: World Health Organization; 2016. 28079998

[10] Moller A-B, Petzold M, Chou D, Say L. Early antenatal care visit: a systematic analysis of regional and global levels and trends of coverage from 1990 to 2013. The Lancet Global Health. 2017;5(10):e977–e83. doi: 10.1016/S2214-109X(17)30325-X 28911763 PMC5603717

[11] Arsenault C, Jordan K, Lee D, Dinsa G, Manzi F, Marchant T, et al. Equity in antenatal care quality: an analysis of 91 national household surveys. The Lancet Global Health. 2018;6(11):e1186–e95. doi: 10.1016/S2214-109X(18)30389-9 30322649 PMC6187112

[12] Benova L, Tunçalp Ö, Moran AC, Campbell OMR. Not just a number: examining coverage and content of antenatal care in low-income and middle-income countries. BMJ global health. 2018;3(2):e000779. doi: 10.1136/bmjgh-2018-000779 29662698 PMC5898334

[13] Lebanese Ministry of Public Health. Service delivery guidelines for reproductive health care. 2016.

[14] Hodgins S D’Agostino A. The quality–coverage gap in antenatal care: toward better measurement of effective coverage. Global Health: Science and Practice. 2014;2(2):173–81. 10.9745/GHSP-D-13-00176 PMC4168625 25276575

[15] Amoakoh-Coleman M, Klipstein-Grobusch K, Agyepong IA, Kayode GA, Grobbee DE, Ansah EK. Provider adherence to first antenatal care guidelines and risk of pregnancy complications in public sector facilities: a Ghanaian cohort study. BMC pregnancy and childbirth. 2016;16(1):1–10. doi: 10.1186/s12884-016-1167-6 27881104 PMC5121950

[16] DeJong J, Akik C, El Kak F, Osman H, El-Jardali F. The safety and quality of childbirth in the context of health systems: mapping maternal health provision in Lebanon. Midwifery. 2010;26(5):549–57. doi: 10.1016/j.midw.2010.06.012 20691519 PMC2989442

[17] Benage M, Greenough PG, Vinck P, Omeira N, Pham P. An assessment of antenatal care among Syrian refugees in Lebanon. Conflict and health. 2015;9(1):1–11. doi: 10.1186/s13031-015-0035-8 25741381 PMC4349304

[18] Talhouk R, Mesmar S, Thieme A, Balaam M, Olivier P, Akik C, Ghattas H, editors. Syrian refugees and digital health in Lebanon: Opportunities for improving antenatal health. Proceedings of the 2016 CHI Conference on Human Factors in Computing Systems; 2016.

[19] Tappis H, Lyles E, Burton A, Doocy S. Maternal health care utilization among Syrian refugees in Lebanon and Jordan. Maternal and child health journal. 2017;21(9):1798–807. doi: 10.1007/s10995-017-2315-y 28707099

[20] El Kak F, Chaaya M, Campbell O, Kaddour A. Patterns of antenatal care in low-versus high-risk pregnancies in Lebanon. EMHJ-Eastern Mediterranean Health Journal, 10 (3), 268–276, 2004. 2004. 16212201

[21] Ghaferi AA, Schwartz TA, Pawlik TM. STROBE reporting guidelines for observational studies. JAMA surgery. 2021;156(6):577–8. doi: 10.1001/jamasurg.2021.0528 33825815

[22] Giles M, Mason E, Muñoz F, Moran A, Lambach P, Merten S, et al. Antenatal care service delivery and factors affecting effective tetanus vaccine coverage in low-and middle-income countries: Results of the Maternal Immunisation and Antenatal Care Situational analysis (MIACSA) project. Vaccine. 2020;38(33):5278–85. doi: 10.1016/j.vaccine.2020.05.025 32527598 PMC7342001

[23] Lu P-j, Srivastav A, Amaya A, Dever JA, Roycroft J, Kurtz MS, et al. Association of provider recommendation and offer and influenza vaccination among adults aged≥ 18 years–United States. Vaccine. 2018;36(6):890–8. 29329685 10.1016/j.vaccine.2017.12.016

[24] Awad DC, Zaiter A, Ghiya P, Zaiter K, Louka JG, Fakih C, et al. COVID-19: Pregnant Women’s Knowledge, Perceptions & Fears. First National Data from Lebanon. Obstetrics and Gynecology Research. 2020;3(4):220–34.

[25] Tafere TE, Afework MF, Yalew AW. Providers adherence to essential contents of antenatal care services increases birth weight in Bahir Dar City Administration, north West Ethiopia: a prospective follow up study. Reproductive health. 2018;15(1):1–8. 30268132 10.1186/s12978-018-0610-8 PMC6162936

[26] Bintabara D, Nakamura K, Ntwenya J, Seino K, Mpondo BC. Adherence to standards of first-visit antenatal care among providers: A stratified analysis of Tanzanian facility-based survey for improving quality of antenatal care. PloS one. 2019;14(5):e0216520. doi: 10.1371/journal.pone.0216520 31083696 PMC6513091

[27] Arroyave L, Saad GE, Victora CG, Barros AJ. Inequalities in antenatal care coverage and quality: an analysis from 63 low and middle-income countries using the ANCq content-qualified coverage indicator. International journal for equity in health. 2021;20(1):1–10. 33865396 10.1186/s12939-021-01440-3 PMC8052706

[28] Raatikainen K, Heiskanen N, Heinonen S. Under-attending free antenatal care is associated with adverse pregnancy outcomes. BMC public health. 2007;7(1):1–8. doi: 10.1186/1471-2458-7-268 17900359 PMC2048953

[29] Billah SM, Ali NB, Khan ANS, Raynes-Greenow C, Kelly PJ, Siraj MS, et al. Factors influencing quality nutrition service provision at antenatal care contacts: Findings from a public health facility-based observational study in 21 districts of Bangladesh. PloS one. 2022;17(1):e0262867. doi: 10.1371/journal.pone.0262867 35085319 PMC8794200

[30] Castrogiovanni P, Imbesi R. The role of malnutrition during pregnancy and its effects on brain and skeletal muscle postnatal development. Journal of Functional Morphology and Kinesiology. 2017;2(3):30.

[31] Imdad A, Bhutta ZA. Effect of balanced protein energy supplementation during pregnancy on birth outcomes. BMC public health. 2011;11(3):1–9. doi: 10.1186/1471-2458-11-S3-S17 21501434 PMC3231890

[32] Ota E, Hori H, Mori R, Tobe‐Gai R, Farrar D. Antenatal dietary education and supplementation to increase energy and protein intake. Cochrane database of systematic reviews. 2015(6). doi: 10.1002/14651858.CD000032.pub3 26031211 PMC12634316

[33] UNICEF. Synthesis of the crisis impact on Lebanon. 2022. [Available from: https://www.unicef.org/lebanon/reports/synthesis-crisis-impact-lebanon.

[34] Save the Children. Enonomic crisis combined with COVID-19 is pushing Lebanon towards a hunger crisis. 2021. [Available from: https://www.savethechildren.org/us/charity-stories/lebanon-economic-hunger-crisis.

