# Supplementary material for: An assessment of the content of antenatal care provided by obstetricians in Lebanon: A cross-sectional study
Source: PLOS Glob Public Health. 2024 Nov 4;4(11):e0003853. doi: 10.1371/journal.pgph.0003853 (PMC11534237; doi:10.1371/journal.pgph.0003853)
Supplement: S2 Table — (DOCX) [file pgph.0003853.s003.docx]

**S2 Table: Adherence of selected antenatal care services by different payment methods in Lebanon.**

|  | **Out-of-pocket payment (n=99 (73.9%))** | | **Other payment methods* (n=35 (26.1%))** | | **P-value** |
| --- | --- | --- | --- | --- | --- |
|  | n | (%) | n | (%) |  |
| **Do you provide tetanus vaccine or DTP?** |  |  |  |  |  |
| No | 74 | (74.7) | 19 | (54.3) | 0.024 |
| Yes | 25 | (25.3) | 16 | (45.7) |  |
| **Do you provide flu vaccine?** |  |  |  |  |  |
| No | 27 | (27.3) | 11 | (31.4) | 0.639 |
| Yes | 72 | (72.7) | 24 | (68.6) |  |
| **Do you provide COVID-19 vaccine?** |  |  |  |  |  |
| No | 17 | (17.2) | 4 | (11.4) | 0.422 |
| Yes | 82 | (82.8) | 31 | (88.6) |  |
| **Do you provide full blood count?** |  |  |  |  |  |
| No | 2 | (2.0) | 1 | (2.9) | 0.999^f^ |
| Yes | 97 | (98.0) | 34 | (97.1) |  |
| **Do you provide group B Streptococcus screening test?** |  |  |  |  |  |
| No | 12 | (12.1) | 7 | (20.0) | 0.251 |
| Yes | 87 | (87.9) | 28 | (80.0) |  |
| **Do you provide blood pressure measurements?** |  |  |  |  |  |
| No | 3 | (3.0) | 0 | (0.0) | 0.567^f^ |
| Yes | 96 | (97.0) | 35 | (100.0) |  |
| **Do you provide midstream urine flow test?** |  |  |  |  |  |
| No | 8 | (8.1) | 5 | (14.3) | 0.323^f^ |
| Yes | 91 | (91.9) | 30 | (85.7) |  |
| **Do you perform at least 5 ultrasounds during pregnancy?** |  |  |  |  |  |
| No | 16 | (16.2) | 11 | (31.4) | 0.053 |
| Yes | 83 | (83.8) | 24 | (68.6) |  |
| **Do you recommend iron?** |  |  |  |  |  |
| No | 9 | (9.1) | 5 | (14.3) | 0.759^f^ |
| Yes | 90 | (90.9) | 30 | (85.7) |  |
| **Do you recommend folic acid during pre-conception and the first trimester?** |  |  |  |  |  |
| No | 7 | (7.1) | 4 | (11.4) | 0.477^f^ |
| Yes | 92 | (92.9) | 31 | (88.6) |  |
| **Do you screen your patients for gestational diabetes by ordering the 1-hour glucose challenge test?** |  |  |  |  |  |
| No | 19 | (19.2) | 5 | (14.3) | 0.515 |
| Yes | 80 | (80.8) | 30 | (85.7) |  |
| **Number of visits recommended** |  |  |  |  |  |
| Less than 8 visits | 33 | (33.3) | 12 | (34.3) | 0.918 |
| 8 or more visits | 66 | (66.7) | 23 | (65.7) |  |
| **Adherence to guidelines** |  |  |  |  |  |
| Adherence to all | 10 | (10.1) | 5 | (14.3) | 0.537^f^ |
| Adherence to some | 89 | (89.9) | 30 | (85.7) |  |

* Other payment methods include: Ministry of Public Health, private insurance, social security, UNHCR or UNRWA

^f^ Fisher’s exact test was used
