# Supplementary material for: An assessment of the content of antenatal care provided by obstetricians in Lebanon: A cross-sectional study
Source: PLOS Glob Public Health. 2024 Nov 4;4(11):e0003853. doi: 10.1371/journal.pgph.0003853 (PMC11534237; doi:10.1371/journal.pgph.0003853)
Supplement: S3 Table — (DOCX) [file pgph.0003853.s004.docx]

**S3 Table:** **Overall and module-specific score of obstetricians’ adherence by area, payment method, and healthcare facility type.**

|  | **Governorates** | | | **Payment method** | | | **Healthcare facility type** | | |
| --- | --- | --- | --- | --- | --- | --- | --- | --- | --- |
|  | **Mont Lebanon or Beirut** | **Other governorates** | **P-value** | **Out-of-pocket** | **Other** | **P-value** | **Clinic in private hospital or private doctor clinic** | **Clinic in public hospital or primary healthcare center** | **P-value** |
|  | Median  [IQR] | Median  [IQR] |  | Median  [IQR] | Median  [IQR] |  | Median  [IQR] | Median  [IQR] |  |
| **Overall score (n=134)** | 77.8  [71.1 ; 80] | 77.8  [68.9 ; 82.2] | 0.632 | 77.8  [71.1 ; 80] | 77.8  [66.7 ; 84.4] | 0.635 | 77.8  [70 ; 80] | 77.8  [64.4 ; 82.2] | 0.682 |
| **Sub score for all respondents (n=134)** |  |  |  |  |  |  |  |  |  |
| Dietary interventions | 100  [80 ; 100] | 100  [80 ; 100] | 0.247 | 100  [80 ; 100] | 100  [60 ; 100] | 0.146 | 100  [80 ; 100] | 100  [60 ; 100] | 0.658 |
| Dietary supplements | 87.5  [75 ; 100] | 87.5  [75 ; 100] | 0.601 | 87.5  [75 ; 87.5] | 87.5  [75 ; 87.5] | 0.556 | 87.5  [75 ; 87.5] | 87.5  [75 ; 100] | 0.399 |
| Antenatal care assessment | 80  [73.3 ; 86.7] | 80  [66.7 ; 86.7] | 0.306 | 80  [73.3 ; 86.7] | 86.7  [73.3 ; 86.7] | 0.596 | 80  [73.3 ; 86.7] | 73.3  [66.7 ; 86.7] | 0.208 |
| Fetal growth assessment | 75  [62.5; 87.5] | 75  [62.5; 87.5] | 0.490 | 75  [62.5; 87.5] | 75  [62.5; 87.5] | 0.371 | 75  [62.5; 87.5] | 75  [62.5; 87.5] | 0.983 |
| Antenatal care preventive measures | 66.7  [55.5 ; 77.8] | 66.7  [55.5 ; 77.8] | 0.490 | 66.7  [55.5 ; 77.8] | 66.7  [55.5 ; 77.8] | 0.371 | 66.7  [55.5 ; 77.8] | 66.7  [55.5 ; 77.8] | 0.984 |
| **Sub score for respondents who provide care to undernourished pregnant women (n=44)** |  |  |  |  |  |  |  |  |  |
| Dietary interventions | 66.7  [66.7 ; 66.7] | 66.7  [66.7 ; 66.7] | 0.725 | 66.7  [66.7 ; 66.7] | 66.7  [66.7 ; 66.7] | 0.561 | 66.7  [66.7 ; 66.7] | 66.7  [66.7 ; 66.7] | 0.681 |
